# Draft Genome Sequence of an Isolate of Extensively Drug-Resistant Mycobacterium tuberculosis from Nepal

**DOI:** 10.1128/MRA.01404-19

**Published:** 2020-01-23

**Authors:** Sanjay S. Gautam, Kelvin W. C. Leong, Manoj Pradhan, Y. Ibotomba Singh, Sagar K. Rajbhandari, Gokarna R. Ghimire, Krishna Adhikari, Uma Shrestha, Raina Chaudhary, Gyanendra Ghimire, Sundar Khadka, Ronan F. O’Toole

**Affiliations:** a School of Medicine, College of Health and Medicine, University of Tasmania, Hobart, Tasmania, Australia; b School of Molecular Sciences, College of Science, Health and Engineering, La Trobe University, Victoria, Australia; c Department of Microbiology, College of Medicine, Nepalese Army Institute of Health Sciences, Kathmandu, Nepal; d National Tuberculosis Control Center, Bhaktapur, Nepal; e HIV Reference Unit, National Public Health Laboratory, Kathmandu, Nepal; f Trinity College Dublin, Department of Clinical Microbiology, Saint James’s Hospital, Dublin, Ireland; University of Arizona

## Abstract

Extensively drug-resistant (XDR) Mycobacterium tuberculosis has become a challenge to the treatment of tuberculosis (TB) in several countries, including Nepal. Here, we report for the first time the draft genome sequence of an isolate of XDR-TB collected in Nepal and describe single-nucleotide variations associated with its extensively drug-resistant phenotype.

## ANNOUNCEMENT

Nepal experienced approximately 42,000 cases of tuberculosis (TB) in 2018, equating to a national incidence rate of 151 per 100,000 individuals (1). Key challenges to TB control in Nepal include finding and treating so-called “missing cases” that are not registered through the National Tuberculosis Control Program (NTP) (2) and overcoming the prevalence of drug resistance. In Nepal in 2017, 2.2% of new cases and 15% of previously treated cases of TB were multidrug resistant (MDR) or rifampin resistant (RR), including 13 laboratory-confirmed cases of extensively drug-resistant (XDR) TB (3). Of particular concern are the relatively low treatment success rates of 68% and 61% observed in Nepal for MDR/RR-TB and XDR-TB, respectively (1).

We previously characterized XDR isolates of Mycobacterium tuberculosis using whole-genome sequencing (4–6). In this work, an XDR-TB isolate from Nepal, NP1701X, was grown in pure culture on Löwenstein-Jensen medium, and genomic DNA was prepared as previously described (7, 8). DNA libraries were generated using the Nextera XT library preparation kit (catalog number FC-131-1024; Illumina, USA) as described previously (9). Default parameters were used for all software unless otherwise specified. Sequencing of the isolate using an Illumina MiSeq instrument produced a total of 1,138,854 paired-end reads which mapped to the publicly available annotated genome of M. tuberculosis reference strain H37Rv (GenBank accession number NC_000962.3) (10) by Burrows-Wheeler alignment (11). This yielded an average read depth of 37.22-fold, covering 99.35% of the H37Rv genome. Variants relative to the H37Rv reference genome were called, and annotation was performed using Geneious Prime 2019.2.3. Variant calling was established using a minimum nucleotide variant frequency of 95% and a minimum sequence read depth of 20. A 4,326,340-bp draft genome assembly of 166 contigs (≥500 bp) was assembled *de novo* using the SPAdes assembler (v3.7) (12). Assembled contigs were ordered with respect to the M. tuberculosis H37Rv genome using ABACAS (13).

The NP1701X isolate belongs to the Beijing sublineage of East Asian lineage 2, as predicted by the PhyResSE and TB Profiler databases (14, 15). A total of 1,352 variant sites were identified in NP1701X relative to the H37Rv genome and consisted of 1,267 single-nucleotide variants (SNVs), 64 insertions/deletions, and 21 substitutions (of 2 or more adjacent nucleotides). A total of 791 of the variants were nonsynonymous, of which 725 were SNVs, 46 were insertions/deletions, and 20 were substitutions. The NP1701X genome displayed high-confidence single-nucleotide polymorphisms, as defined by Feuerriegel et al. (14), that are known to relate to antimicrobial drug resistance in M. tuberculosis based on clinical and experimental data. These include mutations in the *rpoB* gene (tCg/tTg, Ser450Leu), *fabG1-inhA* promoter (t-8c), *pncA* gene (gCc/gTc, Ala134Val), and *embB* gene (Atg/Gtg, Met306Val), which underlie M. tuberculosis resistance to the first-line drugs rifampin, isoniazid, pyrazinamide, and ethambutol, respectively (16–18). A further mutation was found in the *rpsL* gene (aAg/aGg, Lys43Arg) which is related to streptomycin resistance (17). Additional mutations detected in the *gyrA* gene (Tcg/Ccg, Ser91Pro) and *rrs* gene (A1401G) are associated with resistance to fluoroquinolones and second-line injectables (amikacin, kanamycin, and capreomycin), respectively (18). The identification of the latter mutations is in agreement with the extensively drug-resistant phenotype of the NP1701X isolate in culture. This study represents the first published genome sequence assembly of an XDR-TB isolate from Nepal and highlights the potential of using next-generation sequencing for drug resistance detection for medical laboratory diagnostics and surveillance.

### Data availability.

This whole-genome shotgun project has been deposited at DDBJ/ENA/GenBank under the accession number WJSJ00000000. The version described in this paper is the first version, WJSJ01000000. The associated BioProject, SRA, and BioSample accession numbers are PRJNA587824, SRP231411, and SAMN13219581, respectively.
